# Quality of life implications for elevated trait impulsivity in people with Parkinson’s disease

**DOI:** 10.1007/s11136-022-03321-w

**Published:** 2023-01-13

**Authors:** Ashani Jeyadevan, Megan Bakeberg, Michelle Byrnes, Jade Kenna, Sarah McGregor, Soumya Ghosh, Malcom K. Horne, Rick Stell, Tess Evans, Frank L. Mastaglia, Ryan Anderton

**Affiliations:** 1 Faculty of Medicine, School of Nursing and Midwifery, and Health Sciences, University of Notre Dame Australia, Fremantle, WA Australia; 2 Centre for Neuromuscular and Neurological Disorders, University of Western Australia, Nedlands, WA Australia; 3 Perron Institute for Neurological and Translational Sciences Nedlands, Nedlands, WA Australia; 4 Centre for Clinical Neurosciences and Neurological Research, St Vincent’s Hospital Melbourne, Fitzroy, VIC Australia; 5 Florey Institute for Neuroscience and Mental Health, University of Melbourne, Parkville, VIC Australia; 6 Institute of Health Research, University of Notre Dame Australia, Fremantle, WA Australia

**Keywords:** Impulsivity, Quality of life, Parkinson’s disease, Non-motor symptoms

## Abstract

**Background:**

Several non-motor features of Parkinson’s disease (PD) are known to adversely affect patient health-related quality of life (HRQL). However, the specific impact of neuropsychiatric complications, such as impulsive behaviour, is yet to be elucidated.

**Objectives:**

The present cross-sectional, observational study aimed to investigate the effects of heightened trait impulsivity on HRQL in individuals with PD.

**Methods:**

A total of 322 people with idiopathic PD were sequentially recruited from Movement Disorder clinics across Australia. Trait impulsivity in patients was determined by Barratt’s Impulsiveness Scale Version 11 (BIS-11), and grouped into tertiles (low, medium, and high). Patient HRQL was determined by the 39-item Parkinson’s Disease Questionnaire (PDQ-39), complemented by the Cambridge Behavioural Inventory-Revised (CBI-R) indicating caregivers’ perception of patient HRQL.

**Results:**

When total BIS-11 scores were grouped into tertiles, patient perceived and caregiver-perceived HRQL were 1.7-fold (*p* < .001) and 2.2-fold (*p* < .001) worse in the high BIS-11 group when compared to patients in the low group. Univariate analysis revealed significant associations between second-order attentional (*p* < .001) and non-planning (*p* < .001) impulsivity domains with PDQ-39 scores. When controlling for confounding demographic and clinical variables, a multivariate linear regression model revealed second-order attentional impulsivity was independently predictive of poor patient perceived HRQL (*p* < .001).

**Conclusion:**

These findings suggest that increasing trait impulsivity is significantly associated with patient perceived HRQL in PD. Improved knowledge and recognition of subclinical impulsivity may guide clinicians’ treatment and reduce disease burden for patients experiencing PD symptoms.

## Introduction

Parkinson’s disease (PD) is a chronic progressive neurodegenerative disease, which is typified by an assortment of symptoms. These include cardinal motor symptoms of tremor, bradykinesia, rigidity, and postural instability. Research has identified that several non-motor symptoms (NMS) are typically present prior to the onset of cardinal motor signs including cognitive impairment and neuropsychiatric disturbances [1]. Common neuropsychiatric symptoms include impulsive behaviours, depression, anxiety, apathy, psychosis, and sleep disturbances [2]. Neuropsychiatric symptoms often amplify disability with subsequent implications for patient management and outcome. Consequently, NMS have been shown to have a considerable effect on the health-related quality of life (HRQL) of individuals with PD and the greatest burden placed on carers [3]. Health-related quality of life (QoL) is defined as “the patient’s own perception and self-evaluation regarding the effects of an illness and its consequences on his or her life” [4]. Furthermore, neuropsychiatric symptoms in particular have been found to have one of the biggest influences on HRQL and are a significant predictor of institutionalization at an advanced disease stage [2, 5]. Despite this, identifying and managing neuropsychiatric complications in people with PD (PwP) remains a challenge.

Impulsivity is a complex construct, consisting of overly risky, prematurely expressed or generally inappropriate behaviours and actions, which may result in harmful consequences to individuals [6]. This character trait exists on a spectrum, with mild symptoms often overlooked as personality changes, in some cases enhancing creativity and positively contributing to patient functionality [7]. More severe symptoms influence decision making, manifesting as poor planning, discounting future rewards, risk preference and executive dysfunction [7, 8]. These indications of impulsiveness have all been individually correlated to poor HRQL in varying groups of patients with neurodegenerative disorders specifically [9]. Research has revealed that increased impulsivity is concurrently seen in many neuro-compromised patients such as those with Huntington’s Disease [10], traumatic brain injury [11], and ADHD [12].

However, impulsivity itself is a remarkably difficult human behaviour to quantify, often attributed to dopamine agonist use, mild cognitive impairment, or other underlying clinical factors in PwP [13, 14]. Many studies have also noted challenges when differentiating between clinically relevant symptoms and non-pathological behaviour [15–17]. Whilst many previous studies focus on clinical outcomes, self-report measures have been thought to best relate to real life outcomes, therefore most clinically relevant. Amongst PwP, high levels of self-reported trait impulsivity are generally observed [15, 18, 19], this is notable as it represents a significant risk factor for development of Impulse Control Disorders (ICDs) [8]. ICDs consist of performing maladaptive behaviours repetitively, excessively, and impulsively, often to the extent that interferes in the HRQL of oneself and those around them.

Clinically diagnosable ICDs represent the upper end of the impulsivity severity scale, affecting one in seven PwP [8]. ICDs are known to be the primary cause of devastating social consequences such as bankruptcy, divorce, and in some cases, criminal conviction [8]. The ICD group of “behavioural addictions” is extremely heterogeneous and can include pathological gambling, compulsive shopping, binge eating, hypersexuality, and the compulsive misuse of dopaminergic medication [8]. Existing literature has investigated the influence of diagnosed ICDs on HRQL scales in PD cohorts and found that ICDs, particularly in subscales of emotional wellbeing, are an independent predictor of disability, caregiver burden, and poor QoL [15]. Thus, the importance of elucidating specific impact preclinical symptoms may have is clear.

Given the complexity of categorising these preclinical behaviours, the relationship between impulsivity and health related HRQL in PwP remains unexplored. Therefore, the present study investigated this retrospectively in a cohort of Australian PwP. Trait impulsivity was examined by the Barratt’s Impulsiveness Scale 11 (BIS-11), a scale that indicates where patients sit on this spectrum, from unproblematic behaviours to diagnosed ICDs. This multifaceted self-reported questionnaire is designed to encompass subscales of attentional impulsiveness (an inability to concentrate); motor impulsiveness (a tendency to act without thinking), and non-planning impulsiveness (a lack of future planning). Patient HRQL was assessed utilising patient and caregiver perspectives; the Parkinson’s Disease Questionnaire (PDQ-39) and Cambridge Behavioural Inventory – Revised (CBI-R). It was thought that patients with elevated trait impulsivity, across all individual subscales, would have poorer HRQL.

## Methods

### Participants

Participants were recruited into the Australian Parkinson’s Disease Registry between 2012 and 2019. A total of 392 were sequentially recruited from Movement Disorders Clinics across Australia. Clinics were situated at the Perron Institute for Neurological and Translational Science (Perth, Western Australia), St Vincent’s Hospital (Melbourne, Victoria), and the Royal North Shore Hospital (Sydney, New South Wales), as previously described [20, 21]. Of this cohort, 322 home-based PwP and 316 caregivers completed the appropriate clinical measures and were recruited into this study. All patients were confirmed to have idiopathic Parkinson’s disease by a movement disorders neurologist, as per the UK Brain Bank criteria prior to inclusion in the study. All PwP previously diagnosed with an ICD were excluded from this study. At the time of all assessments, patient response to medication was at optimum levels (“ON” period). Written informed consent was obtained from all participants under the National Health and Medical Research Council guidelines. Human Research and Ethics approval was granted from Sir Charles Gairdner Hospital (Approval number 2006/073), and the University of Notre Dame Australia (Approval number 016005F), in accordance with the National Statement on Ethical Conduct in Human Research.

### Clinical assessments

All consenting patients underwent clinical evaluation wherein age, gender, medication status and date of diagnosis were recorded. Patient deep brain stimulation (DBS) history was also noted. PD medications were converted into total levodopa equivalent doses as previously described [22]. The Movement Disorders Society Unified Parkinson’s Disease Rating Scale (MDS-UPDRS) was administered to assess extent of disease in patients. Notably, part III of this assessment (MDS-UPDRS III) was used to determine patient motor symptoms during “ON” periods. Global cognitive function was evaluated using the Scales for Outcomes in Parkinson’s Disease-Cognition (SCOPA-Cog), a screening tool for assessing and differentiating memory and learning, attention, executive function, visuospatial function, and memory domains in PwP [22].

#### Impulsivity evaluation

The validated 30-item Barratt Impulsiveness Scale Version 11 (BIS-11) questionnaire was used to assess the personality and behavioural construct of impulsiveness [23]. It is important to note that BIS-11 cannot be used as a diagnostic tool, however, indicates where individuals sit on this spectrum, from unproblematic behaviours to diagnosed ICDs. BIS-11 has also been used to determine trait impulsivity in PwP [24]. The BIS-11 consists of 30 questions scored on a four-point scale (rarely/never, occasionally, often, almost always/always). The BIS-11 contains questions regarding everyday behaviours, such as whether a patient “plans tasks carefully” or “changes jobs” frequently. Overall BIS-11 scores were calculated as the sum of these 30 scores (to yield a score out of 120), with higher scores indicating greater impulsivity. The sum of second-order BIS-11 attentional, motor, and non-planning items were used to calculate patients BIS-11 s-order domain scores. These second-order scores represent subtypes of impulsiveness, differentiating the complex and varying manifestations of patient presentations. BIS-11 s-order attentional scores were scored out of 32, and motor and non-planning scores both scored out of 44. During the screening process patients displaying ICD symptoms, who did not have a formal diagnosis, were invited to seek further consultation with a clinical psychologist.

#### Patient quality of life evaluation

Patient HRQL was primarily assessed using the Parkinson’s Disease Questionnaire (PDQ-39), a widely used disease-specific health status questionnaire, with adequate internal consistency (Cronbach’s alpha: 0.59–0.94) and retest reliability demonstrated in PD cohorts [25]. The PDQ-39 is a 39-item questionnaire comprising indices of patient physical, mental, and social wellbeing, according to disease symptoms in the preceding month [26]. A caregiver’s perception of patient HRQL was also assessed by the validated Cambridge Behavioural Inventory—Revised (CBI-R) [25, 26]. The CBI-R is an informant based 45-item questionnaire assessing a range of affective, behavioural, and cognitive symptoms in individuals with neurodegenerative brain diseases [27]. The frequency of events for both the PDQ-39 and CBI-R was scored on a five-stage scale ranging from 0 (‘never’) to 4 (‘always’ or ‘cannot do at all’). Poorer HRQL is determined by higher scores in both PDQ-39 and CBI-R measures, with the highest possible score of 156 and 180, respectively.

### Statistical analysis

Data were analysed using IBM-SPSS (v. 26, IBM Corporation), and presented as mean ± standard deviation (SD), unless otherwise stated. A significant nominal *p*-value of < 0.05 was employed. When evaluating the effects of trait impulsivity, BIS-11 scores were treated as both a continuous and grouped variable. The grouped patient BIS-11 scores were separated into tertiles (low, medium, and high), generated using SPSS, for ease of comparison prior to continuous analysis. These three BIS-11 groups were compared to HRQL measures using non-parametric Kruskal Wallis analysis with post hoc corrections due to non-gaussian distribution. Multivariable linear models investigating patient HRQL included impulsivity second-order subscales as independent variables; clinical variables seen to significantly associate with HRQL in univariate analysis, such as DBS treatment history, MDS-UPDRS III motor scores, SCOPA-Cog and disease duration, were also incorporated into models. Non-significant variables were sequentially removed until all factors within the model were significant predictors of PDQ-39 score.

## Results

### Cohort information

Clinical assessment and demographic details are summarised in Table 1. The average age of all 322 participants was 64.3 (± 9.16) years, of which 63.0% were male. Within this heterogenous PD cohort, 45 patients were classified as young onset (under 45 years) [27–29], with a mean disease duration of 8.42 (± 5.66) years. Mean HRQL scores as determined by the patient (PDQ-39) and by the carer (CBI-R) were 32.5 (± 23.1) and 15.7 (± 20.2), respectively.

**Table 1:** Clinical characteristics of the current cohort of patients with Parkinson’s disease

Variable		Mean (SD) or *n* (%)
Gender		
Male		203 (63.0%)
Female		119 (37.0%)
Patient age (years)		64.3 (9.2)
Disease duration (years)		8.42 (5.66)
Total levodopa (mg/day)		848 (599)
DBS		
Yes		37 (11.9%)
No		274 (88.1%)
MDS-UPDRS III		20.2 (13.8)
SCOPA-Cog		28.2 (7.6)
PDQ-39		32.5 (23.1)
CBI-R		15.7 (20.2)
BIS-11		
Mean		60.2 (9.8)
Second-order attentional		15.4 (3.5)
Second-order motor		21.2 (3.7)
Second-order non-planning		23.5 (5.4)

*DBS* deep brain stimulation; *MDS-UPDRS III* movement disorder society—unified Parkinson’s disease rating scale part III; *SCOPA-cog* scales for outcomes in Parkinson’s disease—cognition; *PDQ-39* 39-item Parkinson’s disease questionnaire; *CBI-R* cambridge behavioural inventory-revised; *BIS-11* Barratt’s impulsiveness scale version 11

### Trait impulsivity associates with patient quality of life

**Fig. 1:**
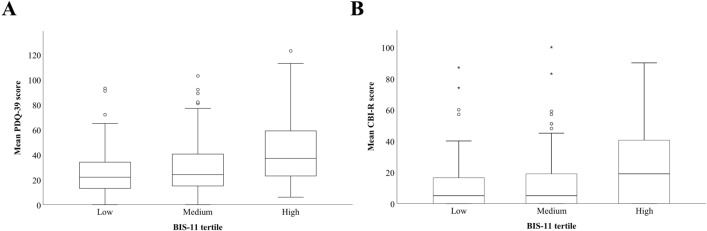
Total BIS-11 impulsivity scores are associated with patient and caregiver HRQL. **A** Within the high range total BIS-11 impulsivity group, mean PDQ-39 scores were significantly elevated when compared to medium and low BIS-11 impulsivity groups. **B** Similarly, within the high range total BIS-11 impulsivity patient group, mean CBI-R scores were significantly elevated when compared to medium and low BIS-11 impulsivity groups

To investigate the effects of trait impulsivity on HRQL, patient BIS-11 scores were separated into tertiles (low, medium, and high) as stated previously. When grouped, mean PDQ-39 scores were significantly different between impulsivity groups (*p* < 0.05). Mean PDQ-39 scores increased 1.7-fold (*t* = 17.46, *p* < 0.001) between low to high impulsivity tertile groups (Fig. 1A). When examining caregiver-perceived HRQL, successive BIS-11 tertiles exhibited increasing CBI-R scores, with a significant increase in CBI-R scores in the highest impulsivity tertile group when compared to the normal and low impulsivity groups (*p* < 0.05). CBI-R scores were increased twofold (*t* = 12.03, *p* < 0.001) from normal to high groupings and 2.2-fold (*t* = 13.31, *p* < 0.001) comparing low to high groupings (Fig. 1B).

### Trait impulsivity subscales associate with patient quality of life

Subsequent generalised linear models investigating PDQ-39 were explored with individual BIS-11 s-order domain scores (Table 2). Regression models revealed second-order attentional (*p* < 0.001; *ß* coefficient = 1.969) and non-planning impulsivity (*p* < 0.001; *ß* coefficient = 1.315) scores were both significantly associated with PDQ-39 scores. In contrast, second-order motor impulsivity was not associated with PDQ-39 scores (*p* = 0.301; *ß* coefficient = 0.366).

**Table 2:** Univariate linear regression models of PDQ-39 (patient perceived quality of life) using BIS-11 s-order subscales

Linear regression models with BIS-11 s-order attentional				
	PDQ-39			
	ß coefficient	Std. Error	*t*	*p*
BIS-11 s-order attentional	1.969	0.355	5.546	< .001*
Constant	2.115	5.624	0.376	0.707
Linear regression models with BIS-11 s-order motor				
	PDQ-39			
	ß coefficient	Std. Error	*t*	*p*
BIS-11 s-order motor	0.366	0.353	1.036	0.301
Constant	24.793	7.587	3.268	0.001
Linear regression models with BIS-11 s-order non-planning				
	PDQ-39			
	ß coefficient	Std. Error	*t*	*p*
BIS-11 s-order non-planning	1.315	0.230	5.726	< .001**
Constant	1.578	5.546	0.285	0.776

*PDQ-39* 39-item Parkinson’s disease questionnaire; *BIS-11* Barratt’s impulsiveness scale version 11

**p < 0.01

### Attentional impulsivity is a significant determinant of patient quality of life

In order to further assess the association between impulsivity and HRQL, generalised linear models (GLM) were constructed to ascertain predictors of patient perceived HRQL. Potential confounding factors, both demographic and clinical variables, were included in the models. Univariate analysis revealed PDQ-39 had strong associations with disease duration (*p* < 0.001), and MDS-UPDRS III motor scores (*p* < 0.001), but not with gender (*p* = 0.983). Medication targeting motor symptoms, specifically total levodopa scores (*p* < 0.001) and DBS (*p* < 0.001), were also seen to associate with PDQ-39 scores significantly. The final model was seen to significantly account for variance in PDQ-39 scores (*X*^2^ = 109.27, *p* < 0.001; Table 3). Following sequential removal of non-significant variables, attentional impulsivity (p = 0.001), total levodopa (*p* = 0.018), MDS-UPDRS III motor scores (*p* < 0.001), DBS (*p* = 0.007), and SCOPA-Cog (*p* = 0.001) remained in the model, with significant influence on PDQ-39 scores. Subsequently, this model indicated for every point increase in the BIS-11 attentional score, PDQ-39 scores were predicted to rise by 1.15 points.

**Table 3:** Final multivariate linear regression parameters of PDQ-39 score

Variable		*ß* coefficient	Std. Error	*p*
Intercept		11.96	7.39	0.106
Second-order attentional		1.15	0.340	0.001**
DBS				
No		−9.75	3.60	0.007**
Yes		0^a^	–	–
MDS-UPDRS III		0.621	0.094	< .001***
Total levodopa (mg)		0.005	0.002	0.018*
SCOPA-Cog		−0.520	0.155	0.001**

*PDQ-39* 39-item Parkinson’s disease questionnaire; *DBS* deep brain stimulation; *MDS-UPDRS III* movement disorder society – unified Parkinson’s disease rating scale part III; *SCOPA-cog* scales for outcomes in Parkinson’s disease – cognition

^a^Comparison category set to zero

*p < 0.05 **p < 0.01 ***p < 0.001

## Discussion

This study examined the influence of trait impulsivity on health-related quality of life (HRQL) in PD. It has been documented that heightened impulsivity is observed in PwP when compared to healthy controls; however, impulsive behaviours often only come to clinical attention after a crisis point has been reached [14, 18, 28]. Despite this, the influence of heightened impulsivity on a patient’s HRQL is yet to be investigated. The current study revealed high levels of trait impulsivity, both as a total score and within subscales, had a significant negative impact on patient HRQL. Specifically, from a patient’s perspective, a stepwise decline in HRQL was found to correspond with an increase in total BIS-11 scores. Previous studies have reported that impulsive traits are predictive of poor HRQL in patients with bipolar disorder, substance abuse and ICDs [15, 29, 30]. We extend on such findings to illustrate this relationship within a large cross-sectional cohort of PwP, with no prior history of ICDs.

This study revealed that all second-order subscales demonstrated a consistent trend of decline in patient perceived HRQL. Previously documented literature has reported that PwP perform poorly in measures of response inhibition, which is a central facet of motor impulsiveness, the tendency to act without thinking [31]. While our results lend support to this notion, there were no statistically significant differences between motor impulsivity groups. In consideration of this, it is important to highlight that in cohorts of patients with diagnosed movement disorders, difficulty arises in differentiating between characteristic uncontrolled movements and motor impulsivity. We suggest that our findings may not have reached statistical significance due to an inability to distinguish between motor impairment characteristic of PD and motor impulsiveness, particularly when suffering from cognitive dysfunction that often occurs in PwP. Future research should assess motor impulsiveness more specifically, prior to analysing its impact on HRQL; tasks such as the Stop Signal Task, the Go/No-Go and the anti-saccade may allow for a clearer distinction between movement disorder and motor impulsiveness.

In addition, this study found that increasing non-planning impulsivity was associated with a stepwise decline in patient perceived HRQL. Non-planning impulsivity manifests in patients as impulsive decision making, with a preference towards instantaneous, larger rewards [32]. Lack of foresight is a common symptom in PD-ICD cohorts, especially those with compulsive gambling and compulsive medication use symptoms [33]. In such conditions, poor HRQL is thought to relate to experiences of burdensome financial losses, legal problems, and inability to disclose difficulties to friends and family [34]. Given this tendency to cause social, occupational, and financial problems, it is anticipated that general mental health may also decline [35, 36]. Prior research and the results of this study revealed that a poor sense of future planning can significantly impact several facets of an individual’s wellbeing and HRQL. Thus, it is important for clinicians to consider non-planning impulsiveness when treating patients, as improving this facet of impulsiveness will potentially improve HRQL both directly, and indirectly.

Linear modelling revealed a relationship between PDQ-39 and attentional impulsivity, independent of several contributing factors. This construct of impulsiveness is characterised by a lack of cognitive persistence and inability to tolerate cognitive complexity [37]. A recent longitudinal study illustrated attentional deficits assessed in the context of cognition, to have predictive power in patient HRQL [9]. Within other populations, attentional deficits have been seen to predict problems in everyday functioning and are closely related to depressed moods, vitality, sleep problems, social functioning, and emotional regulation [38–40]. Moreover, past studies in PwP specifically, have tied attentional impulsiveness symptoms to heightened levels of anxiety and risk of falls, which may contribute to declining HRQL [32, 41]. However, it remains to be seen if attentional impulsivity is a result of PD or medication used in the treatment of this disease*.* Thus, it is important that attentional impulsivity is more widely assessed and discussed with clinicians, as potential effects on wellbeing may be increasingly detrimental than is currently recognised.

In the current study, caregivers reported a greater reduction in HRQL as overall impulsivity scores increased, when compared to the perception of the patient. Discrepancies between caregivers’ and patients’ reports may be accounted for by differences in scales used. However, in a cohort of PwP, both CBI-R and PDQ-39 revealed comparable HRQL results between the two measures [42]. Various studies focussing on ICDs found that caregivers report a markedly increased perception of impulsivity-related behaviours, when compared to both patients’ estimation and clinically reported figures [8, 43]. Similarly, in other neurodegenerative cohorts, caregiver reports were strongly influenced by the burden patients’ behaviours had on their own mental state and wellbeing [44]. In the field of PD, related impulsive behaviours are known to have significant negative implications on burden of care [15, 28]. Thus, caregivers may underestimate patient wellbeing due to the substantial consequence that patient impulsiveness may have on their own wellbeing, which may account for the discrepancies between measures of HRQL exhibited in this study.

### Limitations

A number of limitations of the current study must be acknowledged. Firstly, the self-report nature of the BIS-11 may introduce a degree of bias in the gathered responses due to patients often being less inclined to report impulsive tendencies. In addition, as the presence of depression or anxiety was not recorded, the confounding effect of these psychiatric disorders on patient impulsivity and HRQL was not controlled for.

### Conclusion

In the absence of therapeutics available to reverse or slow PD progression, it is important to identify factors that contribute towards HRQL, specifically those not evident from clinical examination. The findings of this present study align with the broader literature, which suggests that behavioural disturbances, alongside characteristic motor disability, are clinically important to HRQL outcomes. Attentional impulsivity, in particular, was seen to be significantly associated with patient perceived HRQL independent of, and more detrimental than, other confounding variables. Therefore, given the burden on patient HRQL, these results warrant further investigation and recognition of subclinical impulsivity.

## Data Availability

The data that support the findings of this study are available from the corresponding author upon reasonable request.
